# High-Quality Gluten-Free Sponge Cakes without Sucrose: Inulin-Type Fructans as Sugar Alternatives

**DOI:** 10.3390/foods9121735

**Published:** 2020-11-25

**Authors:** Urszula Krupa-Kozak, Natalia Drabińska, Cristina M. Rosell, Beata Piłat, Małgorzata Starowicz, Tomasz Jeliński, Beata Szmatowicz

**Affiliations:** 1 Department of Chemistry and Biodynamics of Food, Institute of Animal Reproduction and Food Research of Polish Academy of Sciences, 10-748 Olsztyn, Poland; n.drabinska@pan.olsztyn.pl (N.D.); m.starowicz@pan.olsztyn.pl (M.S.); 2 Food Science Department, Institute of Agrochemistry and Food Technology (IATA-CSIC), Paterna, 46980 Valencia, Spain; crosell@iata.csic.es; 3 Chair of Food Plant Chemistry and Processing, University of Warmia and Mazury in Olsztyn, 10-748 Olsztyn, Poland; beata.pilat@uwm.edu.pl; 4 Department of Chemical and Physical Properties of Food, Institute of Animal Reproduction and Food Research of Polish Academy of Sciences, 10-748 Olsztyn, Poland; t.jelinski@pan.olsztyn.pl; 5 Sensory Laboratory, Institute of Animal Reproduction and Food Research of Polish Academy of Sciences, 10-748 Olsztyn, Poland; b.szmatowicz@pan.olsztyn.pl

**Keywords:** sucrose replacement, cake, dietary fibre, clean label, texture profile, sensory quality, obesity, celiac disease

## Abstract

Due to its structural and organoleptic functions, sucrose is one of the primary ingredients of many baked confectionery products. In turn, the growing awareness of the association between sugar overconsumption and the development of chronic diseases has prompted the urgent need to reduce the amount of refined sugar in foods. This study aimed to evaluate the effect of complete sucrose replacement with inulin-type fructans (ITFs), namely fructooligosaccharide (FOS), inulin (INU) or oligofructose-enriched inulin (SYN), with different degrees of polymerization on the technological parameters and sensory quality of gluten-free sponge cakes (GFSs). The use of ITFs as the sole sweetening ingredient resulted in the similar appearance of the experimental GFSs to that of the control sample. In addition, all GFSs containing ITFs had similar height, while their baking weight loss was significantly (*p* < 0.05) lower compared to the control products. The total sugar exchange for long-chain INU increased the crumb hardness, while the crumb of the GFS with FOS was as soft as of the control products. The sensory analysis showed that the GFS containing FOS obtained the highest scores for the overall quality assessment, similar to the sugar-containing control sponge cake. The results obtained prove that sucrose is not necessary to produce GFSs with appropriate technological parameters and a high sensory quality. Thus, it can be concluded that sucrose can be successfully replaced with ITF, especially with FOS, in this type of baked confectionery product.

## 1. Introduction

Sugar is one of the primary ingredients of many baked confectionery products, including sponge cakes [1]. Due to many functions critical to obtain the desirable structure and organoleptic properties, sucrose is the most commonly used sugar. It imparts a clean, sweet taste appreciated by consumers, and increases the temperature of starch gelatinization and egg protein denaturation, allowing gas bubbles to expand before gel formation [2]. In addition, sucrose provides foam stability and extends the cake shelf-life [1]. When exposed to the high temperature, sucrose degrades to fructose and glucose, which are the reducing sugars participating in the Maillard browning reactions. In turn, ample studies have provided the evidence for the adverse effects of free sugars overconsumption on health, in particular on the risk of development of non-communicable diseases [3], adverse changes in serum lipids and blood pressure [4], and even cancer [5]. Recently, excessive sugar consumption has attracted particular attention from researchers and epidemiologists, who perceive this phenomenon as a major contributor to the rise in obesity prevalence [6,7,8].

The global prevalence of obesity has increased to pandemic proportions [9]. This worrisome trend is progressively reported in subjects suffering from celiac disease (CD), which changes the clinical picture of this disorder. Tucker et al. [10] found that at the time of diagnosis 44% of adult CD were overweight, 13% were obese, whereas only 3% of them were underweight. Besides, endocrine autoimmunity, particularly in type 1 diabetes, is prevalent among CD patients, approximating 5–7% [11]. The strict gluten-free diet (GFD) is the only available and effective CD therapy. However, health-care professionals have problems with the optimal approach to treating CD in type 1 diabetes [12]. A GFD alleviates the clinical symptoms and improves the health and nutritional status of CD patients over time. On the other side, it is less clear if the strict adherence to the GFD is similarly important to asymptomatic CD patients with concomitant type 1 diabetes [13], as there is scarce unbiased evidence regarding the influence of a GFD in patients with both autoimmune diseases. Compared with a conventional diet, a GFD is characterised with a lower content of proteins, essential vitamins (B12, D, and folate), and minerals (iron, calcium, zinc) [14,15]. In turn, numerous studies have provided evidence for nutrient imbalance resulting from the excessive consumption of hypercaloric and hyperlipidemic packaged gluten-free foods [16]. Many gluten-free products are abundant in simple sugars and saturated fats [17], which are added to improve their palatability and texture. In contrast, the GFD has been reported to provide a lower than recommended intake of dietary fibre [18], having a negative health consequence.

The growing awareness of the association between excessive sucrose intake and development of chronic diseases has prompted an urgent need to reformulate foods to reduce the amount of refined sugar. On the other hand, the numerous advantages and favourable functional properties of sucrose make its total replacement a challenge. The 100% sugar removal caused readily detectable losses in the appearance, texture, and mouthfeel of baked confectionery products [19]. The synthetic low-calorie sweeteners have attracted consumers’ attention and became readily available [20]. However, apart from the high-intensity sweetness, they usually do not provide other functionalities of sucrose necessary to make high-quality cakes. In addition, their breakdown products have controversial health and metabolic effects [21,22].

Recently, there has been an increasing consumer interest in foods of superior quality made from natural ingredients providing functional characteristics while having a reduced sugar content and a lower energy value. As sucrose—being a principal cake ingredient—cannot be easily substituted only by intense sweeteners, several studies have explored the application of natural bulking agents, including dietary fibres, in combination with sweeteners in different cake formulations. Psimouli et al. [2] investigated whether oligofructose, polydextrose, and polyols can replace sugar in cake formulations and showed that oligofructose, lactitol, or maltitol exhibited behaviour similar to sucrose in terms of their influence on batter rheology. To evaluate whether steviol glycosides could partially replace sucrose in bakery products, Zahn et al. [23] produced muffins where 30% sucrose was replaced with rebaudioside A together with several fibres and indicated that a mixture of inulin or polydextrose with steviol glycosides resulted in products with characteristics similar to those of the control muffins. Similarly, Gao et al. [24] used inulin (Frutafit IQ, DP_av_ 5–7, Sensus, Roosendaal, The Netherlands) and stevianna as sucrose substitutes in muffin formulation. They pointed out that the replacement of 50% sugar resulted in muffins having texture, firmness, and springiness similar to these of the control products, while the increased additions of stevianna or inulin had a negative effect on muffin firmness.

In order to fulfil the demand for healthier sponge cakes, studies on sucrose replacement/reduction have been carried out and the use of dietary fibres in combination with sweeteners was proposed. Ronda et al. [19] analysed the effects of total sugar substitution with polysaccharides, oligofructose, and polydextrose, in combunation with polyols on the quality of sponge cake. They indicated that the fresh sponge cakes with polyols and oligosaccharides generally had significantly softer crumb than the control ones; however, the use of oligofructose (Raftilose P-95, Orafti Active Food Ingredients, Oreye, Belgium) caused crust darkening and an increase in crumb firmness during storage. A recent study by Garvey et al. [25] has explored the impact of partial (30%; *w*/*w*) sucrose replacement with natural sweetening ingredients, including oligofructose, in sponge cakes and showed that in comparison to control sample, the formula with oligofructose was not significantly different in terms of the liking of colour, odour, flavour, texture, and overall liking.

To successfully exchange sucrose in the already challenging gluten-free system [26] and obtain a desirable structure and organoleptic properties, the sugar replacing ingredient must exhibit the ability to mimic the functionality of sucrose. Due to their sweetness and beneficial technological and health-related properties, inulin-type fructans (ITFs) could be valuable ingredients of gluten-free products [27,28]. Inulin-type fructans are plant natural storage carbohydrates that occur in many edible fruits and vegetables, and in particularly large amounts in the tubers of Jerusalem artichoke (*Helianthus tuberosus*) and chicory (*Cichorium intybus*). They can be divided into long-chain inulin and short-chain fructooligosaccharides (FOS). The length of the chain determines the physicochemical properties of ITFs [29]. Short-chain FOS (DP < 10) are more soluble and sweeter; therefore, they could be used to improve the mouthfeel of low-caloric products [30], while inulin (DP > 10), due to its lower solubility, higher viscosity and thermostability, could be used as a filler and fat-replacer [31]. Investigations showing the use of ITFs in the baked gluten-free products are scarce and mainly focused on gluten-free bread. To the best of our knowledge, this work represents the first study on the ITFs application as natural sugar alternatives in the gluten-free sponge cake formulation. The study aimed to evaluate the effect of total sucrose replacement with the commercial ITFs of different sweetness and degrees of polymerization, namely fructooligosaccharide, inulin, or oligofructose-enriched inulin, on the mixing and pasting batter behaviour and the quality of gluten-free mini-sponge cakes (GFSs) assessed based on selected technological parameters and sensory descriptors.

## 2. Materials and Methods

### 2.1. Ingredients of Gluten-Free Mini-Sponge Cakes

The ingredients used to make gluten-free mini-sponge cakes (GFSs) are shown in Table 1. Potato starch (PPZ “Trzemeszno” Sp. Z o.o., Trzemeszno, Poland), corn starch (HORTIMEX, Konin, Poland), and fresh eggs from the local supermarket were the main components. The remaining ingredients were rapeseed oil “Kujawski” (ZT “Kruszwica” S.A., Kruszwica, Poland), gluten-free baking powder (BEZGLUTEN, Koniusza, Poland), sugar, and salt.

To produce experimental gluten-free mini-sponge cakes, sugar in the control GFS composition was totally replaced with one of the three commercial inulin-type fructans (ITFs), namely fructooligosaccharide (FOS) with DP_av_ 2–8 and 30% sweetness compared to sucrose (Orafti^®^ P95, Beneo, Tienen, Belgium), inulin (INU) with DP_av_ 8–13 and 10% sweetness compared to sucrose (Frutafit HD, Sensus, Roosendaal, The Netherlands), or oligofructose-enriched inulin (SYN), which is a mixture of oligofructose (DP_av_: 3–9) and inulin (DP_av_ ≥ 10) at a specific ratio of 1:1, and ~25% sweetness compared to sucrose (Orafti^®^ Synergy 1, Beneo, Tienen, Belgium), according to product specification.

### 2.2. Preparation of Experimental Gluten-Free Mini-Sponge Cakes

Gluten-free mini-sponge cakes were prepared following the previously developed method [32]. Briefly, egg whites and salt were whisked (2 min) to form a foam in the stainless bowl in the mixer (KitchenAid Professional K45SS, KitchenAid Europa, Inc., Brussels, Belgium). Then, egg yolks and sugar (in the control GFS) or ITFs (in GFSs with ITFs) were added under continuous vigorous mixing (3 min). Subsequently, starches, baking powder, and oil were added and mixed (3 min/minimal speed) to obtain a smooth homogenous batter. The 30 g portions of batter were dosed into paper moulds (diameter: 50 mm, high: 35 mm), that were put on a baking tray (arranged in three rows, each of four GFSs) and baked at 180 °C for 25 min in an electric oven (AB model DC-21, SVEBA DAHLEN, Fristad, Sweden). Baked GFSs were cooled for 1 h at the room temperature, then packed in a clip-on polyethylene bags, and stored at room temperature pending further analysis. The baking weight loss and height determinations, and instrumental colour analysis of GFSs were performed after cooling (1 h at room temperature), while texture profile and sensory analysis were performed on GFSs stored for 1 day under the described conditions.

### 2.3. Pasting Behaviour of Batters for Gluten-Free Mini-Sponge Cakes over Heating-Cooling Cycles Determined with the Rapid Visco Analyser (RVA)

The viscosity of batters for gluten-free mini-sponge cakes over heating-cooling cycles was evaluated using a Rapid Visco Analyser (RVA-4800; Perten Instruments, Madrid, Spain). The GFS batters were prepared according to the formula presented in Table 1, with the exception that fresh eggs were replaced with whole egg powder (EPSA Additivos Alimentarios, Valencia, Spain) to avoid the variability that the fresh eggs could introduce in a collaborative project. The 8 g batter samples were dispersed in distilled water (12 mL). The obtained suspensions were stirred for 1 min at 600 rpm at 30 °C. After that time, the temperature rose to 95 °C at a rate of 12 °C min^−1^. The sample was maintained for 30 s at 95 °C, cooled to 50 °C at a rate of 12 °C min^−1^, and finally maintained for 2 min at 50 °C. The onset temperature (°C), peak temperature (°C), peak viscosity (PV, cP), hot paste viscosity (HPV; cP), breakdown (PV-HPV; cP), cold paste (final) viscosity (CPV; cP), and setback (CPV-HPV; cP) were recorded. The experiments were conducted in triplicate.

### 2.4. Characteristics of Gluten-Free Mini-Sponge Cakes

#### 2.4.1. Physical Parameters

The weight of GFSs was measured using a digital balance. The height of GFSs was measured at the highest point of the product using a digital calliper. Baking weight loss (WL) was calculated as the ratio between the weight of batter and the weight of the baked and cooled GFS (Equation (1)):

(1) $$\mathrm{WL}\;\left(\%\right)=\;\frac{\left(a-c\right)\times 100}{a}$$

where:

a—the weight of batter in the mould before baking (g),

c—the weight of baked and cooled GFS (g).

#### 2.4.2. Texture Profile Analysis

Textural properties of experimental GFSs were assessed 24 h after baking. The samples were removed from clip-on bags just before testing. Like in the previous study [32], the GFSs were cut horizontally at the height of the mould to form a flat surface. The texture profile analysis (TPA) was performed on the 2 cm-high lower part whereas the upper part was discarded. Hardness, springiness, gumminess, chewiness, cohesiveness, and resilience were determined using a TA.HD Plus Texture Analyser (Stable Micro Systems Ltd., Godalming, UK) equipped with a 5-kg load cell. The sample was placed centrally under an AACC 36-mm cylinder probe with radius (P/36R). The GFS sample was compressed at a constant rate of 1.0 mm s^−1^ at a distance of 5 mm. The probe holds at this distance for 30 s and then withdraws from the sample and returns to its starting position. Each type of GFSs was tested in six replications.

#### 2.4.3. Instrumental Colour Analysis

The instrumental measurements of the crust and crumb colour of GFSs were made using a HunterLab ColorFlex (Hunter Associates Laboratory, Inc., Reston, VA, USA). The measurements were performed through a 3-cm-diameter diaphragm containing an optical glass. The results were expressed in accordance with the CIELab system. The parameters determined were L* (L* = 0 (black) and L* = 100 (white)), a* (+a* = redness and −a* = greenness), and b* (+b* = yellowness and −b* = blueness). Values were the mean of at least six replicates.

The whiteness index (WI) of the crumb [33] was calculated according to Equation (2):

(2) $$\mathrm{WI}=100\;-\;\sqrt{{\left(100-L^{*}\right)}^{2}+a^{*}{}^{2}+b^{*}{}^{2}}$$

The browning index (BI) of the crust [34] was calculated according to Equations (3) and (4):

(3) $$\mathrm{BI}=\;\frac{100\;\times \;\left(x-0.31\right)}{0.17}$$

where:

(4) $$x=\frac{a^{*}+1.75L^{*}}{5.645L^{*\;}+\;a^{*}-\;3.012b^{*\;\;}}$$

The Δ*E_Lab_* difference between two colours [35] was calculated according to Equation (5):

(5) $$\Delta E_{Lab}=\;\sqrt{{\left(\Delta L^{*}\right)}^{2}+{\left(\Delta a^{*}\right)}^{2}+{\left(\Delta b^{*}\right)}^{2}}$$

#### 2.4.4. Evaluation of Early, Advanced, and Final Stage of the Maillard Reaction

The content of available lysine, as an indicator of the early stage of the Maillard reaction, was determined according to the method described by Michalska et al. [36]. Exactly 50 μL of a sample, 100 μL of *o*-phthaldialdehyde reagent, and 100 μL of water were added to wells and incubated for 3 min (96-well microplate; Porvair Sciences, Norfolk, UK). Then, fluorescence was measured at λ_extinction_ = 340 nm and λ_emmision_ = 455 nm using a microplate reader (Infinite^®^ M1000 PRO, Tecan, Switzerland). The quantitative analysis was performed according to the external standard method, employing a calibration curve of N_α_-acetyl-L-lysine ranging from 10 to 250 μM. The content of free intermediate compounds (FIC) was determined after sample extraction with 6% sodium dodecyl sulfate and then their fluorescence was recorded in a microplate reader (Infinite^®^ M1000 PRO, Tecan, Switzerland) setting at λ_extinction_ = 347 nm and λ_emmision_ = 415 nm. Tryptophan fluorescence (TRP) was measured at λ_extinction_ = 290 nm and λ_emmision_ = 340 nm. Results are expressed as fluorescence intensity (FI) per mg of sample dry matter. The FIC and FAST (fluorescence of advanced MRPs and tryptophan) index were calculated as recently reported by Zieliński et al. [37]. The FAST index data were expressed as a percentage (%). The formation of brown pigments (melanoidins) was estimated as reported in detail by Zieliński et al. [37]. Results were expressed as arbitrary absorbance units. All measurements were performed in triplicate.

#### 2.4.5. Sensory Evaluation

A six-member expert panel (five women and one men) previously selected and trained according to ISO guidelines [38] evaluated the sensory characteristics of experimental GFSs 24 h after baking. The assessors were not CD patients but were familiar with gluten-free products and have been aware of tasting starch-based gluten-free sponge cake. A quantitative descriptive analysis (QDA) [39] was applied to assess the sensory characteristics of the experimental GFSs. Before the analysis, vocabularies of the sensory attributes were developed by the panel in a round-table session, using a standardised procedure [40]. Thirteen attributes were evaluated (Table 2). The assessors evaluated the intensity perceived for each sensory attribute on unstructured graphical scales. The scales were 10 cm long and verbally anchored at each end, and the results were converted to numerical values (from 0 to 10 arbitrary units) by a computer. The experimental GFS samples were coded with a three-digit number and presented to the assessors all together in a random order in transparent plastic boxes. The sensory evaluation was carried out in a sensory laboratory room, which fulfils the requirements of the ISO standards [41], under normal lighting conditions at room temperature. To minimise residual effects, bottled mineral water was suggested to drink between each sample evaluation. The results were collected using a computerised system ANALSENS (IAR & FR PAS, Olsztyn, Poland). GFSs were tested in two replications.

### 2.5. Statistical Analysis

The data reported in all the tables are mean values and standard deviations of triplicate observations unless otherwise stated. The differences between experimental GFSs were analysed by a one-way analysis of variance (ANOVA) with Tukey’s multiple comparison test (*p* < 0.05) using GraphPad Prism version 8.0.0 for Windows, GraphPad Software (San Diego, CA, USA).

## 3. Results and Discussion

### 3.1. Pasting Behaviour of Batters for Gluten-Free Mini-Sponge Cakes

**Figure 1:**
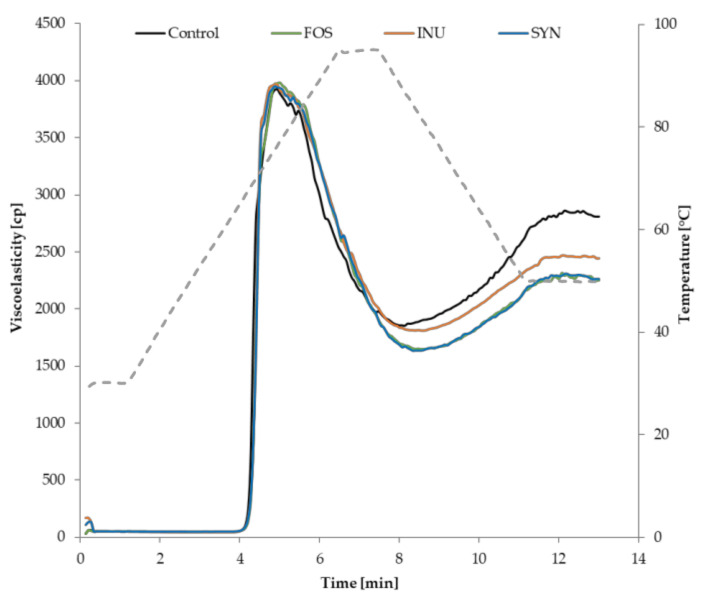
Effect of sucrose replacement with inulin-type fructans (ITFs) on plots of the viscometric profile recorded with the rapid viscoanalyser. FOS—gluten-free mini-sponge cake with FOS; INU—gluten-free mini-sponge cake with inulin; SYN—gluten-free mini-sponge cake with Synergy 1.

The analysis of the pasting properties of gluten-free batters is essential in developing high-quality gluten-free products as it provides information about the changes in paste viscosity behaviour with changes in temperature [32,42]. Changes in the pasting of experimental GFS batters recorded as alterations in batter viscosity due to swelling and pasting of starch granules are shown at the RVA plots (Figure 1). In general, the shapes of RVA plots for the control GFS batter (containing sugar) and batters with ITFs (FOS, INU, SYN) did not differ meaningfully at the mixing and initial heating stage (Figure 1) where all pasting curves were characterised with an initial plateau. During this short period, the viscosity of all experimental GFS batters was similarly low. At this stage, the hydration of potato and corn starch granules took place and increased gradually due to the available water that penetrates into the starch’s interior. The minimal swelling of starch granules could be observed at these temperature conditions (below 50 °C) [43]. Additionally, at the pasting stage, the behaviour of GFS batters could be affected by egg proteins present in the system [44].

Subsequently, as the temperature rose, a sudden increase was recorded in the viscosity of all analysed GFS batters that was observed as a high, sharp, and narrow peak (Figure 1). The starchy ingredients absorbed water available in the batter environment and swelled progressively upon water presence and heat. In all analysed GFS samples, regardless of the presence of sugar (control) or ITFs, excessive expansion of starch granules led to an increase in viscosity up to the maximum apparent viscosity, the so-called peak viscosity (PV). The experimental GFS batters achieved the PV at similar temperature and time (Table 3). Therefore, despite differences in their dextrin chain length, no significant differences were observed in the peak viscosity between the batters. In the next step, when the temperature was constant (95 °C) for 30 s, a substantial reduction in the apparent viscosity of GFS batters was detected (Figure 1) that was determined as the breakdown. Breakdown viscosity is defined as a difference between PV and hot paste viscosity (HPV) and illustrates the ability of the starch to withstand shear stress and heating. Generally, the observed changes were a consequence of the physical breakdown of the starch granules that was accompanied by viscosity decrease. Sugar substitution with ITFs affected this parameter differently; a significant (*p* < 0.05) reduction was determined in HPV (Table 3), particularly when sucrose was exchanged with FOS and Synergy 1. ITFs with intermediate degrees of polymerization led to the higher breakdown; it is likely that those dextrins affect the amylose leaching that accompanied the gelanization process. In the last stage of the RVA analysis, the final viscosity, determined as cold paste viscosity (CPV), and setback were determined. These parameters reflect the ability of the starch polymers to re-organise when the temperature decreases. The setback viscosity of all experimental GFS batters increased (Table 3), that is commonly related to the crystallisation of the amylose chains, but also to the effect of denatured protein [44]. In comparison with the control batter, the value of setback recorded for the batters containing ITFs was significantly (*p* < 0.05) lower (Table 3), suggesting the lower degree of amylose chains crystallisation. The applied ITFs are soluble fibres, however, they differ in chain length, with inulin having the longest chain. That is why the applied ITFs affected the batter pasting characteristics to a different extent.

The study confirmed that the presence of different ITFs in a cake batter modified the pasting profile, particularly after heating and cooling. It has been reported that soluble and insoluble fibres affect the performance of gluten-free layer cakes batter [45], showing that fibres increased the batter viscosity, except for inulin, which decreased it. The present study results even show that the behaviour of the inulins was greatly dependent on their degree of polymerization.

### 3.2. Physical Characteristics and Texture of Gluten-Free Mini-Sponge Cakes

**Figure 2:**
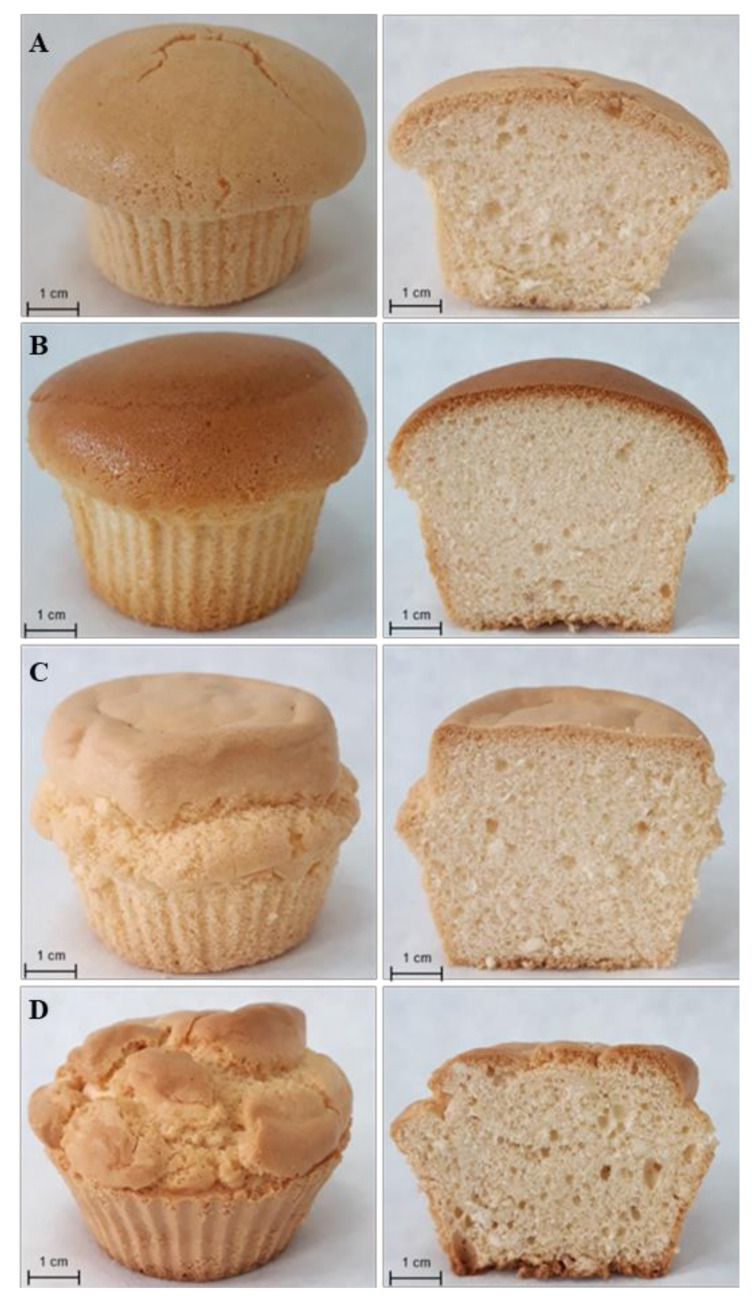
Exemplary pictures of the appearance and cross-section of experimental gluten-free mini-sponge cakes with sucrose replaced with ITFs. (**A**) control gluten-free sponge cake; (**B**) gluten-free sponge cake with FOS; (**C**) gluten-free sponge cake with inulin; (**D**) gluten-free sponge cake with Synergy 1.

The effect of sugar replacement with ITFs on the physical characteristics of GFSs is shown in Table 4 and Figure 2. The experimental sponge cakes containing ITFs were significantly (*p* < 0.05) heavier but similarly high as the control GFS with sugar (Table 4). In the case of foam-type cakes, including particulalry sponge cakes, the high volume and fine porosity are desirable features [1]. The elimination of sucrose did not affect GFSs’ volume. Thus, it appears that ITFs consolidated the structure of experimental GFSs, supported the height, and prevented GFSs’ collapse after baking (Figure 2). The height and final volume of foam-type cakes are mainly determined by gas cell incorporation during mixing and steam production during baking [46]; however, the structure of the cake is established during baking together with the rise of the temperature when the cake matrix solidifies as a result of starch gelatinization and protein denaturation.

All experimental GFSs with ITFs were characterised by significantly (*p* < 0.05) lower baking weight loss in comparison with the control cake containing sugar (Table 4). Baking weight loss is one of the major technological losses and therefore efforts are made to minimise it. Generally, a number of physical and chemical modifications proceed during baking, such as evaporation of water, formation of a porous structure, expansion of volume, etc. [47]. The sponge cake baking process can be divided into the heating up period and crust/crumb period [48]. Baking weight loss results mainly from the drying process [49]; however, water vaporization during the initial heating up period may take place as crust does not appear instantaneously. In addition, other ingredients, ITFs in particular, have a great influence on water retention in baked products. In the case of experimental GFSs without sugar, the decreased value of baking weight loss could be due to ITFs’ ability to bind water molecules [50], causing the higher water retention in the cake during baking. The results obtained by Rodriguez-Garcia et al. [51], where a highly-dispersible native inulin (Frutafit HD^®^, average chain length 8–13, Sensus, Roosendaal, The Netherlands) and highly-soluble oligofructose (Frutafit CLR^®^, average chain length 7–9, Sensus, Roosendaal, The Netherlands) were used indicated that cakes with 50% of native inulin as fat replacer had significantly (*p* < 0.05) lower weight loss than the cakes without it, suggesting that inulins bind water and help to retain moisture during baking.

The effect of sugar replacement with ITFs on textural parameters of the crumb of experimental GFSs is presented in Table 4. The control sponge cake containing sugar had the softest crumb (32.22 N), which at the same time was the most springy and cohesive, and the least gummy and chewy. Sugar replacement with ITFs significantly (*p* < 0.05) affected the TPA profile of the experimental GFSs (Table 4). Inulin increased the hardness of experimental GFSs, while the FOS sample showed crumb softness that was close to the control (37.45 N). Similar observations were made by Gao et al. [24], who revealed that a total replacement of sucrose with inulin (Frutafit IQ, DP_av_ 5–7, Sensus, Roosendaal, The Netherlands) gave muffins with a firmer texture than the control ones. The differences in the action of ITFs applied in the experimental GFSs could be explained by the increase in hardness of inulin gels observed with an increasing degree of polymerisation [52]. In addition, Ziobro et al. [53] demonstrated that the range of changes in textural parameters of the gluten-free bread influenced by ITFs depended on the structure (including DP) and the amount of the applied additives. These authors reported that inulin with a lower DP (HSI with a DP < 10, BENEO-Orafti, Belgium) had a favourable impact on crumb hardness of gluten-free bread, while the loaves with the addition of high performance inulin (HPX) with DP > 23 (BENEO-Orafti, Belgium) were significantly harder. In the case of the remaining texture parameters analysed in the present study, independently of the DP, the complete sugar replacement with ITFs caused rather undesirable changes in the characteristics of GFSs (Table 4). The FOS, INU, and SYN samples were significantly (*p* < 0.05) more gummy and chewy than the control ones, while their springiness and cohesiveness were reduced.

The results of the instrumental colour analysis of crust and crumb of experimental GFSs are shown in Table 4. Compared with the sugar-containing control sponge cake, sugar replacement with ITFs had a significant but different effect on the colour parameters of the crust. Short-chained FOS induced the most pronounced darkening of the crust of the experimental FOS sponge cake (L* = 49.53), followed by SYN. On the other hand, INU in which sugar was replaced with long-chained inulin, the L* value determined for the crust was significantly (*p* < 0.05) lower (L* value = 70.69) compared with the control (64.64) and other GFSs with ITFs. Positive values of coordinates a* (red hue) and b* (yellow hue) were recorded for the crust of all experimental GFSs (Table 4), regardless of the ITFs applied. The results obtained indicated a reddish shade of crust of all GFSs, with the highest value of coordinate a* determined in the FOS sample. The value of the b* coordinate, denoting a yellow shade, was the highest in SYN. The value of the browning index (BI) was inversely related to the crust whiteness; therefore, the highest BI value was recorded for FOS, followed by SYN, whereas the most pronounced reduction in this parameter was recorded for INU (Table 4). Contrary to the crust, the colour of the crumb depends rather on the colour of the ingredients. ITFs used as sugar replacers in the GFS had a similarly white to slightly creamy colour. However, even though no apparent differences were observed in ITFs’ colour, they had an impact on the crumb colour of experimental GFSs. The crumb of the FOS sponge cake was whiter than of the control one (Table 4), while regardless the kind of dietary fibre used in the formulation, crumbs of all GFSs were significantly (*p* < 0.05) redder (a*) and more yellow (b*), compared with the control products. The crumb of the control sponge cake was characterised by the highest whiteness index (WI) (Table 4). In general, the development of brown colour of food that appears during baking is a very typical phenomenon and is mainly caused by non-enzymatic browning reactions which include, among others, caramelisation and Maillard reactions. Carbonyl groups of reducing sugars polymerise with α- and ε-amino groups of proteins, peptides, or amino acids to produce brown nitrogenous pigments (melanoidins) by the spontaneous Maillard reaction [54]. The assessment of the crust colour of the experimental GFSs indicated a pronounced darker colour in the sample containing FOS.

The obtained results of the instrumental colour analysis were consistent with the findings reported by Zahn et al. [31] and suggested that the rate of the Maillard reaction was more intensive in FOS than in other GFSs with ITFs. During the baking process, the hydrolysis of FOS to fructans could occur, thereby increasing the quantity of reducing sugar (especially fructose), promoting the Maillard reaction. The darkening of the crust of experimental GFSs could be perceived as a desirable feature because the gluten-free products generally tend to be paler than their wheat counterparts [26]. In contrast, the light crust of INU may indicate the suppression of the Maillard reaction due to the dilution of the reaction precursor’s in the presence of inulin, a water-retaining ingredient, resulting in a higher water content in the environment [55]. 

Changes in the contents of the early, advanced, and final stage Maillard reaction products affected by sugar replacement with ITFs are presented in Table 5. In this study, the available lysine served as an indicator of the early stage of Maillard reaction, while the fluorescent intermediate products (FIC) formation was considered as the advanced stage of the reaction, and finally, the generation of melanoidins was indicative of the final stage. The FOS sponge cake had available lysine content at the same level as in the control, while that found in INU and SYN was about 20% lower (Table 5). Therefore, the FIC value for control and FOS was similar, whereas the increased amount of FIC was determined in the other GFSs with ITFs. It suggested that INU and SYN promoted the formation of fluorescence compounds, whereas FOS did not. To describe the protein loss in the experimental GFSs, the FAST index was calculated as a ratio between FIC and tryptophan fluorescence presented in%. The lowest value of the FAST index was detected in the control sponge cake (Table 5). Among GFSs with ITFs, a positive effect counteracting proteins loss was noticed in the sample with short-chained FOS, determined as a significantly (*p* < 0.05) lower FAST index percentage. The FAST index values obtained for GFSs were, however, lower than the obtained by Przygodzka et al. [56] for rye-buckwheat cakes enriched with spices. In the GFS containing FOS, an intense form of brown melanoidins was observed (Table 5) that was 45% and 15% higher than in INU and SYN, respectively. The results obtained corresponded well to the results of the instrumental colour analysis (Table 4) and proved that melanoidin formation was positively linked to BI. Nevertheless, except for colour development, many studies demonstrated the health-promoting properties of melanoidins, including their antimicrobial, antioxidant, anti-inflammatory or probiotic effects [57].

**Figure 3:**
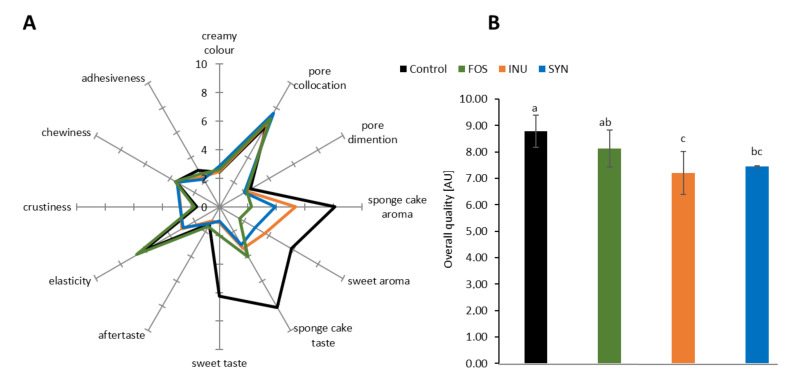
Spider diagram presenting the effect of sucrose replacement with ITFs on QDA parameters (**A**); overall quality (**B**) of gluten-free mini-sponge cakes. FOS—gluten-free mini-sponge cake with FOS; INU—gluten-free mini-sponge cake with inulin; SYN—gluten-free mini-sponge cake with Synergy 1. ^a–c^ Values followed by different letters above the bars are significantly different (*p* < 0.05), as determined by Tukey’s multiple comparisons test.

The acceptance of the sensory quality is essential when a new product is being developed; therefore, an important step in the novel product development is to determine and analyse its quality characteristics, including appearance, aroma, and taste. In the present study, trained experts were asked to assess the experimental GFSs based on their visual appearance (crumb colour and porosity), aroma, taste, and texture, both manually (elasticity) and by the mouth (crustiness chewiness and adhesiveness). The results of QDA are presented in Table 6 and Figure 3. In general, the ITFs used in the formulation did not influence the visual appearance of the crumb of experimental GFSs (Table 6). All GFSs with ITFs looked similar like the creamy-coloured control sponge cake containing sugar. This indicated that the differences in crumb colour detected by the instrumental colour analysis (Table 4) were not perceived by the experts panel in QDA analysis (Table 6). This could be explained by the differences between the methods applied. The instrumental spectrophotometric method makes it possible to define the colour precisely, expressing it numerically in comparison to the standard. The main advantage of this instrumental measurement over the sensory QDA analysis is its higher repeatability resulting from the lower standard deviation due to the lack of variability caused by psychological, physiological, and environmental factors that affect human sensory reactions [58]. The experimental GFSs were similar to the control cakes in terms of porosity features, with both taking into account pore collocation and dimension (Table 6; Figure 2), regardless of the ITFs used in the sponge cake formulation. The number, size, and distribution of air cells incorporated during the mixing stage determine the volume and texture of the baked cakes [1]. A larger number of smaller pores rather than a smaller number of larger ones is a feature of high-quality sponge cakes [32,59]. The control sample was characterised by intensive sponge cake aroma and taste, while in GFSs containing ITFs these features were detected in significantly (*p* < 0.05) lower range, especially in FOS (Table 6). In addition, the experimental GFSs containing ITFs were characterised as having less sweet aroma and taste than the control cakes. Texture evaluation is also an important step in developing a high-quality food product or optimising processing variables.

The ANOVA analysis revealed significant differences (*p* < 0.05) in the QDA texture parameters of the experimental GFSs, in particular in their elasticity (examined manually) and crustiness (assessed in the mouth). In comparison with the control cake, FOS was similarly elastic and had the same crustiness, while INU and SYN were significantly (*p* < 0.05) less elastic and more crusty than the control and FOS (Table 6). The results of sensory analysis corresponded in part with the results of the instrumental texture analysis (Table 4), as both methods indicated a greater similarity of FOS to the control cake than to other GFSs with ITFs. However, the instrumental texture parameters and the sensory descriptors are not defined similarly, while their methodology, including sample size and analysis conditions, are significantly different [60]. When comparing sensory and instrumental analytical methods, it should be noticed that the results of instrumental methods are related to the physical parameters that trigger sensory impressions, while the results of the sensory analysis inform directly about the sensations that these stimuli evoke [61]. Nevertheless, both sensory evaluation techniques and instrumental measurements are equally used to assess texture parameters in food products [62]. In the overall quality assessment, all GFSs containing ITFs were of satisfactory quality, with high scores ranging from 7.21 to 8.13 (Figure 3). However, among experimental GFSs, FOS was favoured and received the highest scores (Figure 2), similar to the control cake containing sugar (8.79). On the other hand, panellists found that the INU and SYN samples were less favoured in the overall acceptance and palatability, compared with the control (*p* < 0.05). These results were in agreement with the results of the instrumental texture and colour analysis (Table 3). The addition of inulin to the experimental sponge cake formulation deteriorated its quality, yielding harder crumb and paler crust (Table 4), and consequently diminishing the sensory quality of GFS. In turn, the use of short-chained FOS improved many technological properties, resulting in the amelioration of the sensorial characteristic of GFS. As was discussed before, the length of the inulin molecule is an important feature affecting the physical and technological properties of the final product. Ziobro et al. [53] reported that the DP of inulin preparations affected the physical characteristics and staling rate of gluten-free bread. Taking into account all gathered results, it could be concluded that a high-quality GFS (i.e., uniform, medium size porosity, proper crumb structure) could be obtained if the appropriate ITFs source was selected based on its functional characteristics, including the DP.

## 4. Conclusions

Our research has demonstrated the feasibility of complete sucrose replacement with ITFs in the gluten-free sponge cake formulation. The use of ITFs as the sole sweetening ingredient resulted in less sweet GFSs which were, however, characterised by a similar appearance to the control sample, especially in terms of the similar crumb colour and porosity. In addition, all GFSs containing ITFs instead of sugar had similar height, while their baking weight loss was significantly lower than in the control cake. The total sugar replacement with ITFs significantly influenced the texture profile of the experimental GFS, in particular the use of long-chain inulin increased the crumb hardness, while the crumb of the GFS with FOS was as soft as that of the control cake. In comparison with the control sample, the GFS containing FOS had a similar content of early Maillard reaction products, determined as the available lysine, which indicated the favourable counteraction of protein loss in this sample. The QDA analysis showed that among the experimental GFSs with ITFs, the sample containing FOS obtained the highest scores for the overall quality assessment, which was similarly high to that given to the sugar-containing control cake. The results obtained prove that sucrose is not necessary to make a gluten-free sugar-free sponge cake with appropriate technological parameters and high sensory quality. Therefore, it can be successfully replaced with ITF, especially FOS, in this type of baked confectionery product.

## Figures and Tables

**Table 1:** Composition of experimental gluten-free mini-sponge cakes.

Ingredient [%]	Control	FOS	INU	SYN
Potato starch	30.6	30.6	30.6	30.6
Corn starch	7.8	7.8	7.8	7.8
Egg	43.0	43.0	43.0	43.0
Sugar	14.0	-	-	-
FOS	-	14.0	-	-
INU	-	-	14.0	-
SYN	-	-	-	14.0
Sunflower oil	3.7	3.7	3.7	3.7
Salt	0.2	0.2	0.2	0.2
Gluten-free baking powder	0.7	0.7	0.7	0.7

FOS—fructooligosaccharides; INU—inulin; SYN—oligofructose-enriched inulin.

**Table 2:** Sensory attributes, their definitions, and scale edges used in the quantitative descriptive analysis (QDA) of gluten-free mini-sponge cakes.

Attribute		Definition	Scale Edges
Appearance	Creamy colour	colour intensity (colour intensity according to colour pattern RAL 075 90 20—scale value 5)	light–dark
	Pore collocation	a visual impression of the arrangement of crumb pores	irregular–regular
	Pore dimension	a visual impression of the size of crumb pores	small–big
Aroma	Sponge cake	the typical odour of sponge cake	none—very intensive
	Sweet	aroma typical of sweet baked products from wheat flour	none—very intensive
Taste	Sponge cake	as the corresponding odour (measured in the mouth)	none—very intensive
	Sweet	basic taste (3% sucrose dissolved in water)	none—very intensive
	Aftertaste	lingering sensation after swallowing the sample	none—very intensive
Texture (manual)	Elasticity	the extent to which a piece of product returns to its original length when pushed by a finger	small–big
Texture (in mouth)	Crustiness	degree of friability released by the sample	small–big
	Chewiness	the multiplicity of chewing the product to prepare it to swallow	low–high
	Adhesiveness	degree of adhesiveness perceived while chewing the sample 10 times	low–high
Overall quality		overall quality including all attributes and their harmonisation	low–high

**Table 3:** Effects of sucrose replacement with ITFs on the Rapid Visco Analyser (RVA) parameters of batter of gluten-free mini-sponge cakes.

	Control	FOS	INU	SYN
Onset temperature (°C) ^1^	64 ± 1 ^ab^	64 ± 1 ^a^	63 ± 1 ^b^	64 ± 1 ^a^
Peak temperature (°C)	77 ± 1	77 ± 1	76 ± 1	77 ± 1
Peak viscosity PV (cP)	3937 ± 16	4014 ± 80	3975 ± 122	3973 ± 54
HPV (cP)	1856 ± 15 ^a^	1648 ± 54 ^b^	1808 ± 43 ^a^	1633 ± 61 ^b^
Breakdown (cP)	2080 ± 1 ^b^	2366 ± 26 ^a^	2167 ± 84 ^b^	2339 ± 15 ^a^
Final CPV (cP)	2846 ± 50 ^a^	2253 ± 68 ^c^	2445 ± 67 ^b^	2265 ± 57 ^c^
Setback (cP)	991 ± 64 ^a^	605 ± 14 ^c^	637 ± 30 ^b^	632 ± 5 ^c^

^1^ Values were presented as mean (*n* = 3) ± standard deviation. ^a–c^ Means with different letters in the same row are significantly different (*p* < 0.05), as determined by Tukey’s multiple comparisons test. HPV—hot paste viscosity; CPV—cold paste viscosity.

**Table 4:** Effects of sucrose replacement with ITFs on physical characteristics and texture of gluten-free mini-sponge cakes.

	Control	FOS	INU	SYN
Weight (g) ^1^	22.45 ± 0.57 ^b^	23.13 ± 0.32 ^a^	23.31 ± 0.13 ^a^	23.23 ± 0.11 ^a^
Height (mm)	49.0 ± 0.52	51.3 ± 0.30	51.0 ± 0.36	48.0 ± 0.10
Baking weight loss (%)	25.18 ± 1.92 ^a^	22.89 ± 1.05 ^b^	22.31 ± 0.44 ^b^	22.58 ± 0.37 ^b^
Textural parameters				
Hardness (N)	32.22 ± 2.49 ^c^	37.45 ± 2.74 ^c^	62.92 ± 3.473 ^a^	51.54 ± 6.639 ^b^
Springiness (%)	0.87 ± 0.03 ^a^	0.79 ± 0.02 ^b^	0.81 ± 0.02 ^b^	0.81 ± 0.03 ^b^
Cohesiveness	0.49 ± 0.01 ^a^	0.33 ± 0.02 ^b^	0.15 ± 0.02 ^c^	0.17 ± 0.03 ^c^
Gumminess	1.60 ± 0.11 ^d^	12.22 ± 0.63 ^a^	10.43 ± 0.90 ^b^	8.56 ± 0.76 ^c^
Chewiness	1.40 ± 0.10 ^c^	9.67 ± 0.66 ^a^	7.70 ± 0.95 ^b^	6.49 ± 0.96 ^b^
Resilience	0.15 ± 0.01 ^a^	0.08 ± 0.01 ^b^	0.05 ± 0.01 ^c^	0.06 ± 0.01 ^c^
Crust colour				
L*	64.64 ± 2.82 ^b^	49.53 ± 0.12 ^d^	70.69 ± 0.95 ^a^	59.71 ± 0.47 ^c^
a*	13.26 ± 0.84 ^c^	17.64 ± 0.20 ^a^	12.09 ± 0.19 ^d^	15.72 ± 0.13 ^b^
b*	35.58 ± 0.95 ^a^	34.14 ± 0.49 ^bc^	33.71 ± 0.54 ^c^	36.09 ± 0.19 ^a^
BI	92.21 ± 5.94 ^c^	133.30 ± 2.56 ^a^	75.66 ± 0.70 ^d^	107.36 ± 1.46 ^b^
*ΔE*		6.44	15.8	5.53
Crumb colour				
L*	82.74 ± 0.58 ^b^	84.01 ± 0.64 ^a^	82.42 ± 0.49 ^b^	81.23 ± 0.92 ^c^
a*	2.01 ± 0.11 ^d^	3.22 ± 0.14 ^b^	2.65 ± 0.14 ^c^	4.59 ± 0.19 ^a^
b*	22.75 ± 0.80 ^c^	24.95 ± 0.64 ^b^	24.15 ± 0.54 ^b^	28.84 ± 0.24 ^a^
WI	71.37 ± 0.65 ^a^	70.18 ± 0.48 ^b^	70.01 ± 0.67 ^b^	65.28 ± 0.62 ^c^
*ΔE*		1.57	2.82	6.79

^1^ Values were presented as mean ± standard deviation. ^a–c^ Values followed by different letters in the same row are significantly different (*p* < 0.05), as determined by Tukey’s multiple comparisons test.

**Table 5:** Effects of sucrose replacement with ITFs on the contents of the early, advanced, and final stage Maillard reaction products in gluten-free mini-sponge cakes.

	Control	FOS	INU	SYN
Available lysine (mg/g) ^1^	12.50 ± 0.42 ^a^	12.51 ± 0.17 ^a^	9.88 ± 0.06 ^b^	9.31 ± 0.09 ^b^
FIC (FI)	28.05 ± 0.60 ^bc^	20.16 ± 0.17 ^c^	50.14 ± 1.94 ^a^	38.56 ± 1.87 ^b^
TRP (FI)	18.00 ± 0.09 ^d^	16.02 ± 0.03 ^c^	20.52 ± 0.78 ^a^	19.44 ± 0.04 ^b^
FAST index (%)	123 ± 5.56 ^c^	175 ± 1.08 ^b^	245 ± 11.28 ^a^	203 ± 19.94 ^b^
Melanoidins (AU)	0.758 ± 0.011 ^b^	0.921 ± 0.012 ^a^	0.504 ± 0.005 ^c^	0.775 ± 0.020 ^b^

FIC—free intermediate compounds; TRP—tryptophan fluorescence; FAST index—fluorescence of advanced MRPs and tryptophan. ^1^ Values were presented as mean ± standard deviation. ^a–c^ Values followed by different letters in the same row are significantly different (*p* < 0.05), as determined by Tukey’s multiple comparisons test.

**Table 6:** Effects of sucrose replacement with ITFs on the sensory quality assessed with a quantitative descriptive analysis (QDA) in gluten-free mini-sponge cakes.

Attribute		Control	FOS	INU	SYN
Appearance	creamy colour	2.42 ± 0.53	2.59 ± 0.62	2.48 ± 0.54	2.86 ± 0.63
	pore collocation	6.59 ± 1.59	7.16 ± 1.87	7.02 ± 0.83	7.56 ± 1.20
	pore dimension	2.51 ± 1.34	2.20 ± 1.95	2.18 ± 0.47	2.03 ± 0.72
Aroma	sponge cake	8.11 ± 1.07 ^a^	2.26 ± 0.85 ^d^	5.34 ± 2.04 ^b^	3.91 ± 1.32 ^c^
	sweet	5.82 ± 1.96 ^a^	1.64 ± 0.92 ^c^	3.69 ± 2.32 ^b^	2.88 ± 1.59 ^bc^
Taste	sponge cake	8.12 ± 0.95 ^a^	4.04 ± 2.31 ^b^	3.34 ± 0.89 ^b^	3.05 ± 1.35 ^b^
	sweet	6.26 ± 1.48 ^a^	1.98 ± 1.20 ^b^	1.09 ± 0.76 ^b^	0.99 ± 0.80 ^b^
	aftertaste	1.39 ± 0.61	1.59 ± 0.96	1.13 ± 0.68	1.31 ± 0.71
Texture (manual)	elasticity	6.18 ± 0.48 ^a^	6.69 ± 1.61 ^a^	3.04 ± 0.92 ^b^	2.92 ± 1.18 ^b^
Texture (in mouth)	crustiness	1.58 ± 0.24 ^b^	1.77 ± 0.53 ^b^	2.72 ± 0.98 ^a^	2.76 ± 0.82 ^a^
	chewiness	3.54 ± 0.44	3.58 ± 0.54	3.36 ± 0.82	3.43 ± 0.93
	adhesiveness	2.98 ± 0.78	2.67 ± 0.67	2.41 ± 0.76	2.24 ± 0.90

Values were presented as mean ± standard deviation. ^a–d^ Values followed by different letters in the same row are significantly different (*p* < 0.05), as determined by Tukey’s multiple comparisons test.
